# HIV pre-exposure prophylaxis use during periods of unprotected sex among female sex workers in Tanga city, Tanzania: a control arm analysis of the pragmatic quasi-experimental trial

**DOI:** 10.3389/fpubh.2024.1405765

**Published:** 2024-07-16

**Authors:** Wigilya P. Mikomangwa, Kåre Moen, Elia J. Mmbaga, Emmy Metta, Stephen M. Kibusi, Melkizedeck T. Leshabari, Appolinary A. R. Kamuhabwa, Gideon Kwesigabo

**Affiliations:** ^1^Department of Epidemiology and Biostatistics, Muhimbili University of Health and Allied Sciences, Dar es Salaam, Tanzania; ^2^Department of Community Medicine and Global Health, University of Oslo, Oslo, Norway; ^3^Department of Clinical Pharmacy and Pharmacology, Muhimbili University of Health and Allied Sciences, Dar es Salaam, Tanzania; ^4^Department of Behavioural Sciences, Muhimbili University of Health and Allied Sciences, Dar es Salaam, Tanzania; ^5^Department of Public Health, The University of Dodoma, Dodoma, Tanzania

**Keywords:** HIV pre-exposure prophylaxis, PrEP prevention-effective adherence, PrEP use, unprotected sex, female sex workers

## Abstract

**Background:**

Pre-exposure prophylaxis (PrEP) prevention-effective adherence is of critical importance but challenging particularly among key populations where periods of high HIV risk are frequent. We assessed the use of PrEP with reference to periods of unprotected sex among female sex workers in the city of Tanga.

**Methods:**

This was part of the pragmatic quasi-experimental trial for HIV PrEP rollout in Tanzania involving a control cohort of 313 female sex workers aged ≥18 years recruited by respondent-driven sampling and followed for 12 months. PrEP use and periods of condomless or unprotected sex were assessed at the 6th and 12th month of follow-up. Prevention-effective adherence was defined as PrEP use of ≥2 pills/week and ≥6 pills/week for anal and vaginal condomless sex. Multivariable modified Poisson regression was conducted to determine factors influencing PrEP use (≥2 pills/week).

**Results:**

Overall, 59.2 and 45.9% of participants had unprotected anal and vaginal sex with a client, respectively. The prevention-effective adherence for anal sex ranged from 8.0% (months 6) to 10.0% (months 12) while that of vaginal sex was from 10.1% (month 6) to 3.8% (month 12). Participants who lived with friends were 25.5 times more likely to use ≥2 PrEP doses per week than those who lived alone (aPR = 25.5; 95%CI: 2.55–255.42, *p* = 0.006). Compared to self-reporting poor health status, self-reporting good health status significantly increased the use of ≥2 PrEP doses per week (aPR = 17.4; 95%CI: 3.01–101.02, *p* = 0.001). Refusing condomless sex with a steady partner increased the likelihood of using ≥2 PrEP doses per week than accepting condomless sex with a steady partner (aPR = 11.2; 95%CI: 1.55–80.48, *p* = 0.017). The prevalence of using ≥2 PrEP doses per week was less among participants accepting condomless sex at high pay than those who refused (aPR = 0.1; 95%CI: 0.03–0.26, *p* = 0.000).

**Conclusion:**

Use of PrEP during periods of unprotected sex was rare among female sex workers. Living with friends, self-reporting good health status, and refusing condomless sex with steady partners were associated with increased use of ≥2 PrEP doses per week. However, accepting condomless sex for increased payment was associated with reduced use of ≥2 PrEP doses per week. This calls for an in-depth study to understand the perspectives and circumstances shaping poor adherence during periods of unprotected sex among female sex workers.

## Introduction

Globally, 1.3 million individuals acquired HIV in 2022 (1). The Sub-Saharan Africa region is most affected by the HIV epidemic, accounting for 50% of global new HIV infections and two-thirds of people living with HIV worldwide: In Tanzania, 1.7 million people were living with HIV in 2022 (1). The main predictor of HIV infection in the general population is unprotected sex with an infected individual who is not adhering to antiretroviral therapy (2). Additionally, multiple sexual partners, early age at sexual debut, forced sex, and sex influenced by drugs or alcohol increase the risk of HIV infection (3).

The HIV infections are more prevalent among members of the key populations and their sexual partners, accounting for 70% of global new infections in 2022 (4). The risk of HIV acquisition is 30 times higher among female sex workers than among women in the general population (5). The disproportionate burden of HIV among female sex workers is attributed to sexual violence, multiple non-regular sexual partners of unknown HIV status, limited access to healthcare, sex influenced by alcohol or drugs, and inability to negotiate condom use especially at high pay (3, 5, 6).

Use of pre-exposure prophylaxis (PrEP) is effective in preventing HIV acquisition among persons at increased risk, including among female sex workers during periods of unprotected sex (7). Due to the interaction in sexual practices between female sex workers and male clients, reducing HIV infections in these groups is likely to significantly reduce new infections.

PrEP is only effective as long as adherence is sufficient during periods of HIV acquisition risk (7). To have protective efficacy, women are required to use ≥2 doses and ≥6 PrEP doses per week for anal sex and vaginal sex, respectively (7–9). Even though female sex workers are at risk of HIV due to sexual exposure, they also experience periods of low or no risk of HIV acquisition, “holidays” during which they do not need to use PrEP, for example during periods of not practicing sex work. Therefore, the use of PrEP should be aligned with periods or seasons of higher risk for HIV acquisition, which change over time (7, 10). At low or no risk, the use of PrEP should be stopped and then restarted when the risk increases, ensuring effective protection to HIV acquisition. This way of taking PrEP is known as prevention-effective adherence (11).

PrEP use during periods of low or no risk is associated with unnecessary burdens and costs to individuals and the health care system, and risks for side effects (12). Promoting PrEP use during periods of high risk for HIV acquisition has the potential to enable optimal use of resources especially in resource-constrained countries. Studies have indicated that individuals do not take PrEP during perceived low-risk periods, and only resume when they perceive they are at high risk of HIV acquisition (1, 4, 5).

Most studies measure PrEP adherence without reference to sexual behavior, making it difficult to determine whether good or poor adherence matches the risk for HIV acquisition (11). Measuring both pill-taking and sexual behavior captures the context of PrEP adherence. Studies that measure pill count, self-reported use of pills or drug concentrations without reference to sexual behavior, cannot explain the disagreement between the level of PrEP adherence and HIV incidence (11). Prevention-effective adherence measures PrEP use together with dynamic socio- or/and sexual behavioral HIV risk indicators (11). Studies conducted among gay men, transgender women, and serodiscordant couples have reported variable levels of prevention-effective adherence ranging from 27% to as high as 99% (12–14). Various individual, structural, societal, and PrEP related factors are attributed to the low use of PrEP (15). However, it is not well known how the use of PrEP matches the sexual behavior of female sex workers. Literature is scarce on the alignment of PrEP use and sexual behavior among female sex workers. Therefore, we examined the use of PrEP during periods of unprotected sex among female sex workers in real-life settings of Tanzania 2 years after countrywide PrEP implementation started.

## Methods

### Study design and setting

This paper reports an analysis of data deriving from the control group of a pragmatic quasi-experimental trial for HIV pre-exposure prophylaxis rollout in Tanzania (PREPTA). The PREPTA study involved two key populations (men who sex with men and female sex workers) in two regions; Dar es Salaam (intervention group) from March 2021 to July 2022 and Tanga (control group) from February 2022 to June 2023. The trial aimed at determining the effectiveness of mobile health technology (mHealth) in optimizing adherence and retention to PrEP among members of the key population (16–18). The project was jointly implemented by the Muhimbili University of Health and Allied Sciences (MUHAS), Tanzania, and the University of Oslo (UiO), Norway. For this paper, an analysis of data was conducted from the cohort of female sex workers in the control region of Tanga to mimic real-life settings of PrEP implementation in Tanzania. The prevalence of HIV among female sex workers in Tanzania is 15.3% (6). The estimated number of female sex workers in Tanga ranges from 7,190 to 9,323 (19, 20). The study was conducted at Ngamiani Health Center, located in the center of the city. Ngamiani Health Center provides PrEP services at a designated building that allows easy and comfortable entry for members of key populations including female sex workers.

### Study population, sampling technique, and follow-up procedures

The study included women who reported having sold sex during the past 3 months preceding the survey and being residents of the Tanga region. The eligibility criteria for PrEP use were as per the Tanzania guidelines (21). Participants were aged ≥18 years, HIV seronegative, and not suspected to have acute HIV infection. They had creatinine clearance >60 mL/min and were consenting to use PrEP as prescribed (8). Participants were recruited through respondent-driven sampling (RDS). The RDS started with three initial participants “seed.” The seeds were recruited strategically to allow for diversity in terms of age, location, and education level. The seeds were selected with the help of peer educators to ensure that the seeds have a large social network. Once the seed completed the interview plus collection of the biological samples, were given the maximum number of three unique coupons for inviting their peers. The use of three coupons ensured that at least 33% of coupons were recovered at the study site. The seed formed the first trend of the sample to be recruited. The recruits of the seed formed the first wave of the recruitment process, then the recruits of wave 1 produced wave 2, and the recruits of wave 2 produced wave 3 etc.: The chaining process continued until the required sample size was reached (16, 18, 22).

Participants were informed by the research team about PrEP use, including information about the meaning of PrEP, its purpose, and importance, as well as refill schedules. Participants were screened for eligibility and prescribed PrEP as per the national guidelines (21). Participants were advised about the need for monthly clinic attendance for PrEP refills. Additionally, they were contacted to participate in research interviews at month 1, month 6, and month 12. The month 1 visit was crucial to determine whether the participants had initiated PrEP and to establish factors hindering the initiation or use of PrEP.

### Sample size

The cohort of all (313) female sex workers in the control group in Tanga was included in the analysis. The sample size of the control group was obtained using a formula for estimating sample size for cohort studies considering the RDS technique of recruitment (23, 24). The sample size was expected to give 80% power to estimate 50% adherence to PrEP with a design effect of 2 even with a 20% potential loss to follow-up.

### Data collection

An electronic Swahili questionnaire was used to collect information since Swahili is Tanzania’s national language and all participants spoke Swahili. The questionnaires were administered by trained research assistants (not female sex workers) and the interview lasted for about 15–25 min. The interviews were conducted at the health center in a designated room which had minimal interference from non-participants. The baseline questionnaire asked about baseline information including demographics, social and sexual behavior characteristics, alcohol use, PrEP awareness, and knowledge about HIV. The follow-up questionnaires (months 1, 6, and 12) asked about PrEP use and sexual behavior: experience of coercive sex, number of sexual partners, type of sex (anal or vaginal), condom use, and sex under the influence of drugs and alcohol. Other information collected included self-perceived HIV risk and PrEP use.

### Variables

#### Outcome variable

To determine prevention-effective adherence, we aligned number of pills used per week and the type of sex engaged (anal or vaginal). To establish the number of pills taken: At month 6 and month 12, participants were asked about the number of pills they had missed in the last 1 month. All participants were dispensed with 30 PrEP pills to cover the entire month (assumed to be 30 days), we subtracted the number of pills missed to get the number of pills used in that month. Since each month is 4 weeks long, we divided the number of pills used per month to get the number of pills used per week, i.e., number of pills used per week = [(30 pills prescribed – number of pills missed) ÷ 4 weeks]. Additionally, at month 12, participants were asked about the number of pills they had used in the past 7 days: Short recall periods such as the previous 7 days can improve the accuracy of the self-reporting (11). The 7-day PrEP use was considered a gold standard to obtain the sensitivity and specificity of deducing the number of pills used from monthly to weekly. The sensitivity and specificity for ≥2 doses per week was 92.0 and 91.4%, with Receiver Operating Characteristic (ROC) of 0.92 (95%CI: 0.86–0.98).

To achieve ≥95% protection, ≥2 doses of PrEP per week are required for anal sex while ≥6 doses of PrEP per week are required for vaginal sex (9). Tenofovir accumulates 10 times more in colorectal tissue than in the female genital tract while emtricitabine accumulates 140 times more in the female genital tract than in colorectal tissue (8, 9). Therefore, we categorized prevention-effective adherence based on the type of sexual practice (anal vs. vaginal). Thus, prevention-effective adherence at 2–5 pills per week (anal sex) and 6–7 pills per week (vaginal sex) considering periods of unprotected sex. We defined periods of unprotected sex as periods when female sex workers practiced condomless sex. The most recent unprotected sex was also assessed by self-report since the use of biomarkers of sexual intercourse such as prostate-specific antigens in vaginal secretions are impractical in real-life clinical settings (11).

#### Independent variables

The independent variables were age, education level, marital status, having children, income from sex work, history of STIs, use of illicit drugs, self-perceived HIV status, PrEP self-efficacy, PrEP awareness, self-reported health status, financial difficulties in accessing health, experience of physical violence and history of being arrested by police. Additionally, social support using a 5-Likert scale of 8 items, was adapted from the Duke-UNC Functional Social Support Questionnaire (FSSQ) and yielded Cronbach’s alpha of 0.88, and a total score below 32 was considered inadequate social support. Nevertheless, alcohol use categories were assessed using the Alcohol Use Disorder Identification Test (AUDIT); a score ≤ 7 (low risk), a score of 8–14 was considered ‘harmful’ or ‘hazardous’ alcohol use, and a score > 14 was defined as alcohol dependence. Furthermore, perceived sex work stigma was measured using 13 items, with five response options each (1 = Strongly disagree, 2 = Disagree, 3 = Neither disagree nor agree, 4 = Agree, 5 = Strongly agree), and high reliability (Cronbach alpha of 0.84). We categorized sex work stigma: Low (scores ≤26); moderate (scores 27–38), and; high (scores ≥39). Also, perceived PrEP stigma was assessed using 10 scale items, with five response options (1 = Strongly disagree, 2 = Disagree, 3 = Neither disagree nor agree, 4 = Agree, 5 = Strongly agree), and Cronbach alpha was 0.88. Categories of PrEP stigma were low (score ≤ 30) and high (score > 30). Lastly, PrEP awareness was measured using 8 true or false questions about PrEP and participants were regarded to have high PrEP awareness if responded correctly to ≥75% of questions and low awareness if scored otherwise (16, 22).

### Data analysis

A descriptive analysis of the periods of unprotected sex is presented as frequencies and proportions of condomless sex. We cross-tabulated PrEP use (three categories: 0–1 dose/week, 2–5 doses/week, and ≥6 doses/week) with periods of unprotected sex (defined as unprotected sex in the most recent sexual encounter) to determine the proportion of individuals who achieved protective doses. Baseline and follow-up characteristics of participants were described using medians with interquartile ranges (IQR) for continuous variables and proportions for categorical variables. As appropriate, Chi-square and Fischer Exact tests were used to test for differences between the proportion of various categorical variables and PrEP use of 0–1 doses/week and ≥2 doses per week. We determined factors associated with the use of ≥2 doses per week of PrEP. Based on the study by Cottrell et al. (9), 65% and ≥95% of the population using 2 pills/week of PrEP achieved protective concentration in the female genital tract and colorectal tissue, respectively. Additionally, the majority (68.0%) of the female sex workers in our study practiced anal sex with or without vaginal sex. Therefore, we used 2 pills/week as the lowest dose possible in determining the factors associated with PrEP use per week. Variables with *p*-value ≤0.2 in bivariate analysis were subjected to a modified Poisson regression model with robust standard errors. The prevalence of the dependent outcome (PrEP use of ≥2 doses/week) was 20.5%, therefore the modified Poisson regression model was preferred to multivariable logistic modeling which could have overestimated the effect of independent variables. Variables at *p*-value <0.05 in multivariable modified Poisson regression modeling were deemed statistically significant determinants of PrEP use of ≥2doses/week. The analysis was conducted using STATA (Version 18).

## Results

### Sociodemographic and structural characteristics by PrEP use

The median age of participants was 27 (IQR: IQR: 23–32) years. A large proportion of the participants were not married (70.6%), had children (85.1%), lived with their family (79.4%), had completed primary education (46.9%), and reported sex work as the only source of income (52.8%). A large majority (76.7%) went to public health facilities when they had health concerns. More than half (52.7%) of the participants had adequate social support, medium sex work stigma (66.3%), and low PrEP stigma (77.4%). Most (73.2%) had high self-perceived risk of contracting HIV, and 4.1% had had a sexually transmitted infection (STIs) during the past 6 months. Nearly three-quarters (73.5%) were alcohol dependent as per AUDIT screening, whereas less than one in five used illicit drugs including cannabis (18.0%). At baseline, more than three-fifths (63.4%) had low PrEP awareness, with high self-efficacy to PrEP (99.5%). At 6 months (*n* = 195) and 12 months (*n* = 189) of follow-up, almost all participants reported to have ever used PrEP (99.5%). At month 6 and month 12, the use of ≥2 doses per week was 20.5 and 19.7%, respectively. PrEP use per week was significantly associated with age (*p* = 0.046), living arrangements (*p* = 0.043), and self-perceived HIV risk (*p* = 0.029) (Table 1).

**Table 1:** Distribution of sociodemographic and structural characteristics by PrEP use among female sex workers.

Variables	All *N* (%)	PrEP doses used per week		*p*-value
		0–1 *n* (%)	≥2 *n* (%)	
Overall	195	155 (79.5)	40 (20.5)	
Age groups (years)				*0.046*
18–24	58 (29.9)	51 (33.1)	7 (17.5)	
25–34	107 (55.2)	78 (50.6)	29 (72.5)	
35+	29 (14.9)	25 (16.2)	4 (10.0)	
Marital status				0.206
Never married	137 (70.6)	112 (72.7)	25 (62.5)	
Married/previously married	57 (29.4)	42 (27.3)	15 (37.5)	
Education level				0.732
No formal education	18 (9.3)	13 (8.4)	5 (12.5)	
Primary	91 (46.9)	73 (47.4)	18 (45.0)	
Secondary+	85 (43.8)	68 (44.2)	17 (42.5)	
Have children				0.325
Yes	165 (85.1)	129 (83.8)	36 (90.0)	
No	29 (14.9)	25 (16.2)	4 (10.0)	
Living arrangements				*0.043^1^*
Alone	19 (9.8)	18 (11.7)	1 (2.5)	
Family	154 (79.4)	123 (79.9)	31 (77.5)	
Friends/others	21 (10.8)	13 (8.4)	8 (20.0)	
Sex work is the only source of income				0.266
No	92 (47.2)	70 (45.2)	22 (55.0)	
Yes	103 (52.8)	85 (54.8)	18 (45.0)	
PrEP awareness				0.087
Low	123 (63.4)	93 (60.4)	30 (75.0)	
High	71 (36.6)	61 (39.6)	10 (25.0)	
PrEP self-efficacy				1.000^1^
Low	1 (0.5)	1 (0.7)	0 (0.0)	
High	192 (99.5)	152 (99.3)	40 (100.0)	
Social support				0.573
Inadequate	87 (47.3)	67 (46.2)	20 (51.3)	
Adequate	97 (52.7)	78 (53.8)	19 (48.7)	
Sex work stigma				0.697
Low	33 (17.6)	25 (16.8)	8 (21.1)	
Moderate	124 (66.3)	101 (67.8)	23 (60.5)	
High	30 (16.0)	23 (15.4)	7 (18.4)	
PrEP Stigma				0.941
Low	147 (77.4)	117 (77.5)	30 (76.9)	
High	43 (22.6)	34 (22.5)	(23.1)	
Self-perceived HIV risk				*0.029^1^*
High risk	142 (73.2)	119 (77.3)	23 (57.5)	
Medium risk	16 (8.2)	10 (6.5)	6 (15.0)	
Low risk	24 (12.4)	17 (11.0)	7 (17.5)	
No risk	7 (3.6)	6 (3.9)	1 (2.5)	
Do not know	5 (2.6)	2 (1.3)	3 (7.5)	
Self-reported health status				0.184^1^
Very good	104 (54.2)	77 (50.7)	27 (67.5)	
Good	70 (36.5)	59 (38.8)	11 (27.5)	
Fair or poor	18 (9.4)	16 (10.5)	2 (5.0)	
Financial difficulties due to spending on health				0.234
Yes	84 (43.3)	70 (45.5)	14 (35.0)	
No	110 (56.7)	84 (54.5)	26 (65.0)	
Reported having had STIs in the past 6 months				0.670^1^
Yes	8 (4.1)	6 (3.9)	2 (5.0)	
No	186 (95.9)	148 (96.1)	38 (95.0)	
Experienced physical violence in the past 12 months				0.952
Yes	78 (40.4)	62 (40.5)	16 (40.0)	
No	115 (59.6)	91 (59.5)	24 (60.0)	
Ever used illicit drugs, including cannabis				0.920
Yes	35 (18.0)	28 (18.2)	7 (17.5)	
No	159 (82.0)	126 (81.8)	33 (82.5)	
Alcohol use (AUDIT)				0.410^1^
Low risk	29 (15.3)	22 (14.8)	7 (17.5)	
Harmful or hazardous	21 (11.1)	19 (12.8)	2 (5.0)	
Alcohol dependence	139 (73.5)	108 (72.5)	31 (77.5)	
Arrested by police in the past 12 months				0.669
Yes	53 (27.3)	41 (26.6)	12 (30.0)	
No	141 (72.7)	113 (73.4)	28 (70.0)	

AUDIT, Alcohol Use Disorder Identification test; PrEP, Pre-Exposure Prophylaxis; STIs, Sexually Transmitted infections. *p*-value by chi-square test; ^1^*p*-value by Fisher’s exact test. The total may not add up due to non-responses to some of the questions. The italicized values indicate variables that are statistically significant i.e. at *p*-value is less than 0.05.

### Sex work characteristics by PrEP use

The median age at first sex and first selling of sex was 17 (IQR: 15–18) years and 20 (IQR: 18–25) years, respectively. More than a third (43.3%) of female sex workers had had sex with more than 30 sexual partners (clients) in the last month. About two-thirds (68.0%) had ever had anal sex with clients, had had sex while drunk (70.9%), and more than a third had used illicit drugs during in the most recent sexual encounter (40.0%). More than half (59.2%) of female sex workers reported having had unprotected anal sex - and 45.9% unprotected vaginal sex – with clients the last time they engaged in these activities, respectively. More than two-thirds (69.4%) of participants said they never used condoms with clients while 1.6, 6.5, and 19.4% said they used condoms “always” “often,” and “sometimes,” respectively. Nearly half (46.4%) refused condomless with a steady partner. More than two-thirds (68.8%) of those who accepted condomless sex for higher pay used ≤1 dose of PrEP per week (*p* = 0.005). A large majority (86.6%) of participants who used ≤1 dose of PrEP per week accepted anal sex for increased payment (*p* = 0.007). Other sex work characteristics were not significantly associated with the use of ≥2 doses of PrEP per week (*p* > 0.05) (Table 2).

**Table 2:** Distribution of sex work characteristics by PrEP use.

Variables	All *N* (%)	PrEP doses per week		*p*-value
		0–1 *n* (%)	≥2 *n* (%)	
Age at sex debut (years)				0.945
<18	127 (65.5)	101 (65.6)	26 (65.0)	
18+	67 (34.5)	53 (34.4)	14 (35.0)	
Age at first selling sex (years)				0.438
<18	31 (16.0)	24 (15.6)	7 (17.5)	
18–24	118 (60.8)	97 (63.0)	21 (52.5)	
25+	45 (23.2)	33 (21.4)	12 (30.0)	
Number of sexual partners last month				0.234
0–9	51 (26.3)	40 (26.0)	11 (27.5)	
10–29	59 (30.4)	43 (27.9)	16 (40.0)	
30+	84 (43.3)	71 (46.1)	13 (32.5)	
Income from sex work per month (TZS^2^)				0.409
≤150,000	38 (19.6)	33 (21.4)	5 (12.5)	
150,001–299,000	42 (21.6)	31 (20.1)	11 (27.5)	
300,000–444,999	66 (34.0)	54 (35.1)	12 (30.0)	
≥450,000	48 (24.7)	36 (23.4)	12 (30.0)	
Have steady partner^¥^				0.858
Yes	80 (41.2)	64 (41.6)	16 (40.0)	
No	114 (58.8)	90 (58.4)	24 (60.0)	
Anal sex with steady partner				0.815
Yes	28 (35.0)	22 (34.4)	6 (37.5)	
No	52 (65.0)	42 (65.6)	10 (62.5)	
Anal sex with clients				0.766
Yes	132 (68.0)	104 (67.5)	28 (70.0)	
No	62 (32.0)	50 (32.5)	12 (30.0)	
The last time you had sex with clients you drank alcohol				0.265
Yes	117 (70.9)	91 (68.9)	26 (78.8)	
No	48 (29.1)	41 (31.1)	7 (21.2)	
The last time you had sex with a client, you used illicit drugs				0.301
Yes	14 (40.0)	10 (35.7)	4 (57.1)	
No	21 (60.0)	18 (64.3)	3 (42.9)	
Accepted condomless sex for more pay				*0.005*
Yes	124 (63.9)	106 (68.8)	18 (45.0)	
No	70 (36.1)	48 (31.2)	22 (55.0)	
Used condom during anal sex with client				0.381
Yes	53 (40.8)	40 (38.8)	13 (48.1)	
No	77 (59.2)	63 (61.2)	14 (51.9)	
Used condom during vaginal sex with client				0.121
Yes	105 (54.1)	79 (51.3)	26 (65.0)	
No	89 (45.9)	75 (48.7)	14 (35.0)	
Accepted anal sex for more pay				*0.007^1^*
Yes	106 (80.3)	89 (86.6)	17 (60.7)	
No	25 (18.9)	14 (13.5)	11 (39.3)	
Forced to have sex in the past 12 months				0.831
Yes	56 (28.9)	45 (29.2)	11 (27.5)	
No	138 (71.1)	109 (70.8)	29 (72.5)	
Refused condomless sex with steady partner				
Yes	89 (46.4)	67 (44.1)	22 (55.0)	0.218
No	103 (53.6)	85 (55.9)	18 (45.0)	
Primary place used most to have sex				1.000^1^
Rented room (Guest house/hotel)	179 (92.8)	142 (92.8)	37 (92.5)	
Others (brothel/Outdoor/home)	14 (7.2)	11 (7.2)	3 (7.5)	

PrEP, pre-exposure prophylaxis; TZS, Tanzanian Shilling. *p*-value by chi-square test; ^1^*p*-value is by Fisher’s exact test. ^2^At the time of writing of the report, 1 United States Dollar was equivalent to 2545.0 Tanzania Shillings (TSh). ^¥^Steady partner includes non-commercial male partner, i.e., boyfriends or husbands. Total may not add up due to none responses to some questions. The italicized values indicate variables that are statistically significant i.e. at *p*-value is less than 0.05.

### PrEP use during the periods of unprotected sex at 6 and 12 months

**Figure 1:**
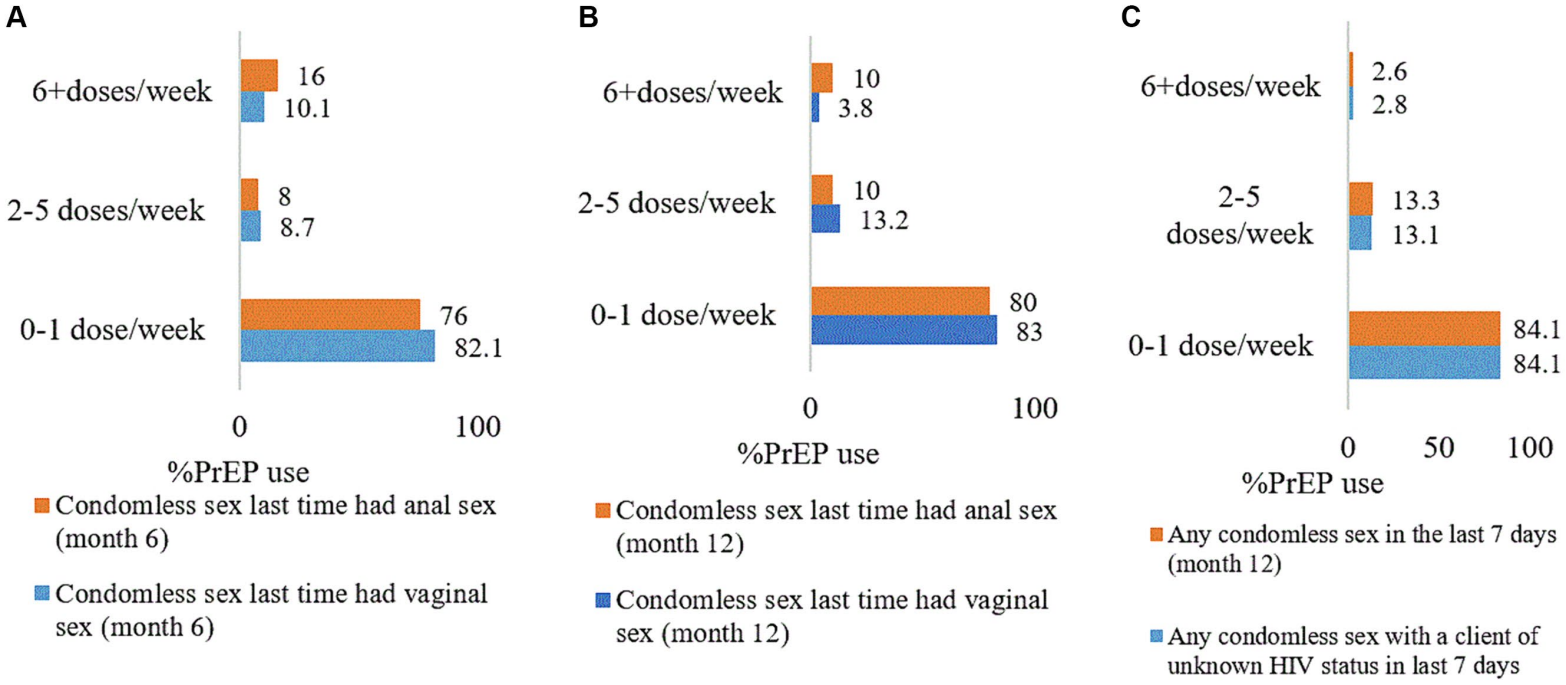
Alignment of unprotected sex by PrEP doses per week among female sex workers. **(A)** Month 6, **(B)** month 12, and **(C)** last 7 days of month 12. The findings in this figure were for participants who reported unprotected sex. PrEP, pre-exposure prophylaxis.

The prevention-effective adherence at month 6 for unprotected anal (2–5 doses/week) and vaginal sex (≥6 doses per week) was 8.0 and 10.1%, respectively. At month 6, most participants had used less than one dose per week during the most recent unprotected anal (76.0%) and vaginal (82.1%) sex (Figure 1A).

At month 12, the prevention-effective adherence among participants who had unprotected anal and vaginal sex was 10.0 and 3.8%, respectively. Moreover, 83.0 and 80.0%, respectively, of those who engaged in unprotected anal and vaginal sex, used less than one dose per week (Figure 1B).

Additionally, 2.6% of female sex workers who had had any unprotected sex (vaginal or anal or both) in the last 7 days preceding the 12-month interview used ≥6 doses per week. A majority (89.5%, *n* = 67) of those who had protected sex did not use any PrEP pill. Nine (9) participants who had not had sex in the last 7 days, had not use any PrEP dose. More than three-quarters (84.1%, *n* = 113) of participants who had condomless sex with clients of unknown HIV status used less than one PrEP dose per week (Figure 1C).

### Determinants of use of ≥2 doses per week of PrEP

Participants living with friends had a higher prevalence of using ≥2 PrEP doses per week than those who lived alone (aPR = 25.5; 95%CI: 2.55–255.42, *p* = 0.006). Those who self-reported good health status had a 17 times higher prevalence of using ≥2 PrEP doses per week than participants who self-reported poor health status (aPR = 17.4; 95%CI: 3.01–101.02, *p* = 0.001). The prevalence of using ≥2 PrEP doses per week was less among participants accepting unprotected/condomless sex at increased payment (aPR = 0.1; 95%CI: 0.03–0.26, *p* = 0.000). The use of ≥2 PrEP doses per week was significantly associated with refusing unprotected/condomless sex with a steady partner (aPR = 11.2; 95%CI: 1.55–80.48, *p* = 0.017) (Table 3).

**Table 3:** Modified poisson regression modeling for determinants of use of ≥2 doses of PrEP per week.

Variables	Crude estimates		Adjusted estimates	
	PR	*p*-value	aPR	*p*-value
Age group (years)				
18–25	Ref			
25–34	2.3 (1.06–4.86)	*0.035*	2.4 (0.48–12.57)	0.283
35+	1.1 (0.36–3.60)	0.820	1.0 (0.13–6.94)	0.977
Marital status				
Married or previously married	1.5 (0.84–2.57)	0.180	0.5 (0.17–1.75)	0.306
Never married	Ref			
Living arrangements				
Family	3.8 (0.55–26.74)	0.173	2.8 (0.39–20.41)	0.308
Friends	7.2 (0.99–52.91)	0.051	25.5 (2.55–255.42)	*0.006*
Alone	Ref			
PrEP awareness				
High	Ref			
Low	1.7 (0.89–3.29)	0.109	0.6 (0.10–3.25)	0.527
Self-perceived HIV risk				
High/medium risk	0.6 (0.33–1.90)	0.097	1.5 (0.31–7.18)	0.610
Low/no risk	Ref			
Self-reported Health status				
Good	1.7 (0.95–3.16)	0.071	17.4 (3.01–101.02)	*0.001*
Poor	Ref			
Accepted condomless sex for more pay				
Yes	0.4 (0.27–0.81)	*0.007*	0.1 (0.03–0.26)	*0.000*
No	Ref			
Accepted anal sex for more pay				
Yes	0.4 (0.20–0.68)	*0.002*	0.9 (0.27–2.84)	0.817
No	Ref			
Condom use with clients				
Yes	Ref			
No	0.5 (0.19–1.08)	0.074	0.4 (0.12–1.48)	0.174
Condom during vaginal sex with client				
Yes	1.6 (0.87–2.80)	0.139	0.9 (0.25–3.52)	0.932
No	Ref			
Refused condomless sex with steady partner^¥^				
Yes	1.4 (0.82–2.49)	0.200	11.2 (1.55–80.48)	*0.017*
No	Ref			

PR, prevalence ratio; aPR, adjusted prevalence ratio. ^¥^Steady partner includes non-commercial male partner, i.e., boyfriends or husbands. The italicized values indicate variables that are statistically significant i.e. at *p*-value is less than 0.05.

## Discussion

We analyzed the use of PrEP in periods of unprotected sex and determined factors associated with the use of ≥2 doses of PrEP per week in a cohort of female sex workers of a PREPTA study in Tanga City, Tanzania. We found that a large majority (76.0–83.0%) of participants who had unprotected vaginal and anal sex used less than one dose of PrEP per week. The trend of prevention-effective adherence between 6 and 12 months for unprotected anal sex varied from 8.0 to 10.0% while that of unprotected vaginal sex varied from 10.1 to 3.8%. The determinants of PrEP use per week were living arrangements, self-reported health status, practicing unprotected sex at increased payment, and refusing unprotected sex with a steady partner.

We found low prevention-effective adherence among female sex workers. Currently, published studies are scarce on prevention-effective adherence among female sex workers resource resource-constrained settings such as Tanzania. Studies conducted elsewhere have found higher prevention-effective adherence in other populations. The Partners Demonstration Project conducted in Kenya and Uganda reports the prevention effective adherence to be as high as 88% (7). A prospective observational study in Taiwan and the EPIC-NSW trial in Australia have also reported high prevention-effective adherence to daily PrEP (14, 25). While our study included only female sex workers, the Partners Demonstration Project involved sero-discordant couples whereas the Taiwan and EPIC-NSW trials involved men who have sex with men. This difference in the study population could have contributed to the differences in prevention effective adherence due to variations in sexual behavior and HIV risk perception between populations. Also, reinforced adherence counseling during follow-ups could have led to higher prevention-effective adherence in those studies compared to our study which did not reinforce adherence counseling during follow-up visits. Additionally, knowing the HIV status of the partner could promote PrEP prevention adherence. For instance in the Partners Demonstration Project, serodiscordant couples knew their partners’ HIV status and were motivated to adhere to PrEP such that some wanted for the relationship to succeed and aimed to have children (7). In our study, most study participants had unprotected sex with clients of unknown HIV status. Despite high self-perceived HIV risk in our study, the prevention effective adherence was very low for both unprotected anal and vaginal sex.

Although poor adherence is common among PrEP users, our findings reveal that poor adherence coincides with periods of sexual behavior that carry a higher risk for HIV acquisition (unprotected/condomless sex). PrEP is not a very efficient HIV prevention strategy if users do not adhere to medication during periods of unprotected sex.

We found that all participants who had not had sex, and most of those who had had protected sex, in the last 7 days preceding the 12-month interview, did not use PrEP pills. This is in line with the prevention-effective adherence paradigm previously reported by Haberer et al. (11). PrEP is only beneficial if adherence coincides with sexual behavior associated with risk for HIV acquisition, such as unprotected sex. However, this approach represents a programmatic challenge to HIV programs due to the lack of validated risk assessment tools that could take into consideration various parameters such as condom use, number of sexual partners, alcohol use, and drug use among others. This makes it difficult even for healthcare providers to advise PrEP users on when to resume PrEP after episodes of discontinuation. In current practice, risk assessments are not conducted routinely in the PrEP program, and this leaves PrEP users alone in making decisions about whether to start or stop PrEP based on self-perceived HIV risk. HIV risk perception has been reported to be one of the strong predictors of PrEP use (26).

We found that female sex workers who lived with friends were more likely to use PrEP compared to those who lived alone. It is well established that family and friends play a significant role in PrEP care, they can either promote or hamper PrEP use (27, 28). The qualitative study of young males and females in South Africa and Uganda cited receiving care, encouragement, and support from friends, immediate partners and family encouraged them to use PrEP (27). Similar findings were echoed in the in-depth interviews involving young men who have sex with men in Thailand which emphasized the role of social relationships and disclosure with significant others in promoting PrEP adherence (29). Studies of adolescent girls and young women in Zimbabwe and South Africa reported that a majority of its participants disclosed PrEP use to friends and that those who disclosed were more likely to adhere than those who did not (30, 31).

We noted that many female sex workers accepted condomless sex with clients at increased payment. More than half (52.8%) of the participants depended solely on sex work as their main source of income therefore, they may have resolved to accept condomless sex regardless of the higher perceived HIV risk. Payment negotiations including higher rates for condomless sex have been reported to be a common practice among sex workers in sub-Saharan countries such as Kenya, South Africa, the Democratic Republic of Congo, and Côte d’Ivoire (32–35). We found that those who accepted condomless sex were less likely to use ≥2 doses of PrEP per week, subjecting them to a higher risk of acquiring HIV. With the currently limited literature on the relationship between PrEP use and accepting condomless sex at high pay, we hypothesize that female sex workers were reluctant to use the PrEP pills for the fear of losing the highly paying client: being perceived by the client as having HIV due to the resemblance of pills and package with antiretroviral medications as we found 22.6% perceived high PrEP stigma: The in-depth interview on this construct will be reported separately. The extent of condom use among female sex workers did not change much over the 12 months of follow up and this may indicate a gap in the PrEP care delivery in Tanzania. Contrary to our findings, a Kenyan study demonstrated increased condom use among PrEP users, associated with comprehensive education on safer sex to PrEP users (36). Accepting condomless sex at increased payment coupled with poor prevention-effective adherence to PrEP places female sex workers at increased risk of HIV and STIs. It has also been reported that PrEP users have an increased incidence of STIs due to condomless sex (37).

Participants who refused unprotected sex with their steady partners were more likely to use ≥2 doses of PrEP per week. This could be attributed to the good understanding of the high HIV risk among the study population. Sex workers perceived as at increased risk of HIV and want to protect their steady partners. It is documented that; male partners of female sex workers have a high prevalence of HIV compared to males in the general population (38). Thus, PrEP and consistent condom use with a steady partner are likely to play an important HIV protection role.

Female sex workers who self-perceived to have good health had a higher prevalence of using ≥2 doses of PrEP per week. There are no studies that have reported PrEP use in relation to self-reported health status. We hypothesize that perceiving a good health status is one of the motivating factors for an individual to use PrEP with the desire to remain negative and be in good health. This could be attributed to the fact that most of them had high self-efficacy with PrEP. Golub et al. (39) reported an increase in PrEP use with self-efficacy. More in-depth information is not in the scope of this paper and will be reported separately.

### Limitations

The study had several limitations. PrEP adherence counseling was conducted routinely by healthcare workers as per the PrEP program implementation, we did not reinforce counseling during follow-up visits. Correspondingly, there was a high attrition rate, 47.6% (1 month), 37.7% (6 months), and 39.6% (12 months) affecting the precision of estimates in the multivariable analysis. Some of the reasons for the high attrition rate were unavailability upon contact via mobile number, migration to other cities for sex work or settlement, stopping sex work, getting married and not being interested in continuing in the study. We have not conducted the loss to follow-up study to quantify the reasons for loss to follow-up. The sexual characteristics including condom use were self-reported which is subject to desirability bias due to the sensitive nature of the behaviors. However, reporting the most recent sexual practice reduced the chances of recall bias. Nevertheless, our study findings provide a good picture of the actual PrEP use practices. Lastly, the use of self-reported adherence or PrEP use could have suffered from social desirability or recall bias. However, the sensitivity and specificity analysis reflected the reduced chances of false negatives and false positives as the result of recall or social desirability biases. Additionally, self-report is the cost-effective approach for assessing adherence that is used in real-life clinical settings.

## Conclusion and recommendations

Use of PrEP during periods of unprotected sex was rare among female sex workers. The use of ≥2 doses per week of PrEP was high among female sex workers with good health status, who refused unprotected sex with a steady partner, and those living with friends. Accepting condomless sex at high pay reduced the use of PrEP among female sex workers. Poor adherence during periods of behavior associated with acquiring HIV reduces the overall benefit of PrEP care in Tanzania. This calls for an in-depth study to better understand the perspectives and circumstances shaping poor adherence during periods of unprotected sex among female sex workers. Further studies should be conducted involving larger samples and with comprehensive documentation of sexual behavior supported with qualitative interviews to further explore the experiences and perspectives of prevention effective adherence among female sex workers. Furthermore, implementation research designed to optimize PrEP adherence, especially at periods with a higher risk of HIV acquisition is of paramount importance.

## Data availability statement

The raw data supporting the conclusions of this article will be made available by the authors, without undue reservation.

## Ethics statement

The studies involving humans were approved by Muhimbili University of Health and Allied Sciences Research Ethics Committee and National Institute for Medical Research (Tanzania). The studies were conducted in accordance with the local legislation and institutional requirements. The participants provided their written informed consent to participate in this study.

## Author contributions

WM: Conceptualization, Data curation, Formal analysis, Investigation, Visualization, Writing – original draft. KM: Conceptualization, Funding acquisition, Methodology, Resources, Supervision, Validation, Writing – review & editing. EJM: Conceptualization, Funding acquisition, Methodology, Resources, Software, Validation, Writing – review & editing. EM: Investigation, Project administration, Resources, Writing – review & editing. SK: Conceptualization, Supervision, Writing – review & editing. ML: Conceptualization, Funding acquisition, Methodology, Project administration, Resources, Writing – review & editing. AK: Conceptualization, Investigation, Supervision, Writing – review & editing. GK: Conceptualization, Data curation, Formal analysis, Investigation, Methodology, Supervision, Validation, Writing – review & editing.

## References

[1] World Health Organization. United Republic of Tanzania HIV country profile 2022. Glob HIV Program Heal Organ. (2022) 2022:1. doi: 10.5089/9798400215414.002

[2] Broyles LN Luo R Boeras D Vojnov L. The risk of sexual transmission of HIV in individuals with low-level HIV viraemia: a systematic review. Lancet. (2023) 402:464–71. doi: 10.1016/S0140-6736(23)00877-2, PMID: 37490935 PMC10415671

[3] Chawla N Sarkar S. Defining “high-risk sexual behavior” in the context of substance use. J Psychosexual Heal. (2019) 1:26–31. doi: 10.1177/2631831818822015

[4] The path that ends AIDS. UNAIDS Global AIDS Update 2023. Geneva: Joint United Nations Programme on HIV/AIDS (2023).

[5] Asefa A Midaksa G Qanche Q Wondimu W Nigussie T Bogale B. Does the perception of HIV risk among female sex workers affect HIV prevention behavior? Application of the health belief model (HBM). BMC Public Health. (2022) 22:1646. doi: 10.1186/s12889-022-14046-3, PMID: 36042424 PMC9427084

[6] Mizinduko MM Moen K Likindikoki S Mwijage A Leyna GH Makyao N. HIV prevalence and associated risk factors among female sex workers in Dar es Salaam, Tanzania: tracking the epidemic. Int J STD AIDS. (2020) 31:950–7. doi: 10.1177/0956462420917848, PMID: 32772690

[7] Haberer JE Kidoguchi L Heffron R Mugo N Bukusi E Katabira E. Alignment of adherence and risk for HIV acquisition in a demonstration project of pre-exposure prophylaxis among HIV serodiscordant couples in Kenya and Uganda: a prospective analysis of prevention-effective adherence. J Int AIDS Soc. (2017) 20:21842. doi: 10.7448/IAS.20.1.21842, PMID: 28741331 PMC5577705

[8] Anderson PL Glidden DV Liu A Buchbinder S Lama JR Guanira JV. Emtricitabine-tenofovir exposure and pre-exposure efficacy in men who have sex with men. Sci Transl Med. (2012) 4:1–17. doi: 10.1126/scitranslmed.3004006 PMC3721979 22972843

[9] Cottrell ML Yang KH Prince HMA Sykes C White N Malone S. A translational pharmacology approach to predicting outcomes of Preexposure prophylaxis against HIV in men and women using Tenofovir Disoproxil fumarate with or without Emtricitabine. J Infect Dis. (2016) 214:55–64. doi: 10.1093/infdis/jiw077, PMID: 26917574 PMC4907409

[10] Elsesser SA Oldenburg CE Biello KB Mimiaga MJ Safren SA Egan JE. Seasons of risk: anticipated behavior on vacation and interest in episodic antiretroviral pre-exposure prophylaxis (PrEP) among a large national sample of U.S. men who have sex with men (MSM). AIDS Behav. (2016) 20:1400–7. doi: 10.1007/s10461-015-1238-0, PMID: 26538056 PMC4854804

[11] Haberer JE Bangsberg DR Baeten JM Curran K Koechlin F Rivet Amico K. Defining success with HIV pre-exposure prophylaxis: a prevention-effective adherence paradigm 11 network for multidisciplinary studies in ARV-based HIV prevention, Lima, Peru 12 Universidad peruana HHS public access. AIDS. (2015) 29:1277–85. doi: 10.1097/QAD.0000000000000647 26103095 PMC4480436

[12] Cooney EE Reisner SL Saleem HT Althoff KN Beckham SW Radix A. Prevention-effective adherence trajectories among transgender women indicated for PrEP in the United States: a prospective cohort study. Ann Epidemiol. (2022) 70:23–31. doi: 10.1016/j.annepidem.2022.03.016, PMID: 35398255 PMC9167788

[13] Gilbert HN Wyatt MA Pisarski EE Timothy R Heffron R Katabira ET. PrEP discontinuation and prevention-effective adherence: experiences of PrEP users in Ugandan HIV Serodiscordant couples. J Acquir Immune Defic Syndr. (2020) 82:265–74. doi: 10.1097/QAI.0000000000002139 PMC6812551 31609925

[14] Bavinton BR Vaccher S Jin F Prestage GP Holt M Zablotska-Manos IB. High levels of prevention-effective adherence to HIV PrEP: an analysis of substudy data from the EPIC-NSW trial. J Acquir Immune Defic Syndr. (2021) 87:1040–7. doi: 10.1097/QAI.0000000000002691, PMID: 33852503

[15] Sidebottom D Ekström AM Strömdahl S. A systematic review of adherence to oral pre-exposure prophylaxis for HIV-how can we improve uptake and adherence? BMC Infect Dis. (2018) 18:581. doi: 10.1186/s12879-018-3463-4, PMID: 30445925 PMC6240194

[16] Mbotwa C Kazaura M Moen K Leshabari M Metta E Leyna G. Predictors of mHealth use in promoting adherence to pre-exposure prophylaxis among female sex workers: an evaluation of the Jichunge intervention in Dar es Salaam, Tanzania. BMC Health Serv Res. (2022) 22:859–12. doi: 10.1186/s12913-022-08245-2, PMID: 35787285 PMC9254514

[17] Lichtwarck HO Mbotwa CH Kazaura MR Moen K Mmbaga EJ. Early disengagement from HIV pre-exposure prophylaxis services and associated factors among female sex workers in Dar es Salaam, Tanzania: a socioecological approach. BMJ Glob Heal. (2023) 8:e013662. doi: 10.1136/bmjgh-2023-013662, PMID: 38154811 PMC10759139

[18] Haaland I Metta E Moen K. The use of PrEP among men who have sex with men and transgender women as biomedical prevention work: a conceptual framework. Soc Sci Med. (2023) 333:116147. doi: 10.1016/j.socscimed.2023.116147 37556992

[19] Dutta A Barker C. Makyao N. Consensus estimates on key population size and HIV prevalence in Tanzania. National AIDS Control Program, Ministry of Health and Social Welfare Tanzania. (2014) 1–34.

[20] Tanzania ministry of health. Tanzania HIV impact survey (this) a population-based HIV impact assessment. Phia (2018); 1–6. Available at: https://phia.icap.columbia.edu/wp-content/uploads/2017/11/Tanzania_SummarySheet_A4.English.v19.pdf

[21] National Aids Control Program, Ministry of Health, Community Development, Gender, Eldery and Children, Tanzania. Implementation framework for pre-exposure prophylaxis of HIV in Tanzania mainland. (2021) 10–5.

[22] Lichtwarck HO Kazaura MR Moen K Mmbaga EJ. Harmful alcohol use and associated socio-structural factors among female sex workers initiating HIV pre-exposure prophylaxis in Dar es Salaam, Tanzania. Int J Environ Res Public Health. (2023) 20:698. doi: 10.3390/ijerph20010698 PMC9819768 36613018

[23] World Health Organization. Regional Office for the Eastern Mediterranean. (2013). Introduction to HIV/AIDS and sexually transmitted infection surveillance: module 4: introduction to respondent-driven sampling. Available at: https://iris.who.int/handle/10665/116864

[24] Kirkwood Betty R. Sterne Jonathan A. C. Essential medical statistics, 2nd Hoboken: Blackwell Publishing Company. (2003). 292–294.

[25] Wu HJ Wen-Wei KS Chang HH Li CW Ko NY Strong C. Imperfect adherence in real life: a prevention-effective perspective on adherence to daily and event-driven HIV pre-exposure prophylaxis among men who have sex with men – a prospective cohort study in Taiwan. J Int AIDS Soc. (2021) 24:1–9. doi: 10.1002/jia2.25733 PMC8138098 34018330

[26] Ssuna B Katahoire A Armstrong-Hough M Kalibbala D Kalyango JN Kiweewa FM. Factors associated with willingness to use oral pre-exposure prophylaxis (PrEP) in a fisher-folk community in peri-urban Kampala, Uganda. BMC Public Health. (2022) 22:468–8. doi: 10.1186/s12889-022-12859-w, PMID: 35264123 PMC8905810

[27] Muhumuza R Ssemata AS Kakande A Ahmed N Atujuna M Nomvuyo M. Exploring perceived barriers and facilitators of PrEP uptake among young people in Uganda, Zimbabwe, and South Africa. Arch Sex Behav. (2021) 50:1729–42. doi: 10.1007/s10508-020-01880-y, PMID: 33954824 PMC8213546

[28] Ongolly FK Dolla A Ngure K Irungu EM Odoyo J Wamoni E. “I just decided to stop”: understanding PrEP discontinuation among individuals initiating PrEP in HIV care centers in Kenya. J Acquir Immune Defic Syndr. (2021) 87:E150–8. doi: 10.1097/QAI.0000000000002625, PMID: 33492024 PMC8026512

[29] Yu YJ Schieber E Janamnuaysook R Wang B Gunasekar A Mac Donell K. Barriers and facilitators to pre-exposure prophylaxis (PrEP) uptake and adherence among men who have sex with men (MSM) in Thailand: a qualitative study. AIDS Care. (2024):1–9. doi: 10.1080/09540121.2024.2332443 PMC12637369 38574278

[30] Beauchamp G Hosek S Donnell D Chan KCG Anderson PL Dye BJ. The effect of disclosure of PrEP use on adherence among African young women in an open-label PrEP study: findings from HPTN 082. AIDS Behav. (2023) 28:1512–21. doi: 10.1007/s10461-023-04175-0 37768427 PMC11069481

[31] Giovenco D Pettifor A Powers KA Hightow-Weidman L Pence BW Edwards JK. The effect of PrEP use disclosure on adherence in a cohort of adolescent girls and young women in South Africa. AIDS Behav. (2022) 26:1007–16. doi: 10.1007/s10461-021-03455-x, PMID: 34478015 PMC8891396

[32] Valente PK Mantell JE Masvawure TB Tocco JU Restar AJ Gichangi P. “I Couldn’t afford to resist”: condom negotiations between male sex workers and male clients in Mombasa, Kenya. AIDS Behav. (2020) 24:925–37. doi: 10.1007/s10461-019-02598-2, PMID: 31321637 PMC6980499

[33] Sikhosana N Mokgatle MM. A qualitative exploration on accounts of condom-use negotiation with clients: challenges and predicaments related to sex work among street-based female sex workers in Ekurhuleni district, South Africa. Pan Afr Med J. (2021) 40:54. doi: 10.11604/pamj.2021.40.54.29918 35059100 PMC8724014

[34] Ntumbanzondo M Dubrow R Niccolai LM Mwandagalirwa K Merson MH. Unprotected intercourse for extra money among commercial sex workers in Kinshasa, Democratic Republic of Congo. AIDS Care. (2006) 18:777–85. doi: 10.1080/09540120500412824 16971288

[35] Becquet V Nouaman M Plazy M Masumbuko JM Anoma C Kouame S. Sexual health needs of female sex workers in Côte d’Ivoire: a mixed-methods study to prepare the future implementation of pre-exposure prophylaxis (PrEP) for HIV prevention. BMJ Open. (2020) 10:e028508–12. doi: 10.1136/bmjopen-2018-028508 PMC6955511 31919122

[36] Manguro GO Musau AM Were DK Tengah S Wakhutu B Reed J. Increased condom use among key populations using oral PrEP in Kenya: results from large scale programmatic surveillance. BMC Public Health. (2022) 22:304–9. doi: 10.1186/s12889-022-12639-6, PMID: 35164707 PMC8842980

[37] Irungu EM Ngure K Mugwanya KK Awuor M Dollah A Ongolly F. “Now that PrEP is reducing the risk of transmission of HIV, why then do you still insist that we use condoms?” the condom quandary among PrEP users and health care providers in Kenya. AIDS Care. (2021) 33:92–100. doi: 10.1080/09540121.2020.1744507 32207327 PMC7511416

[38] Namale G Kamacooko O Kawuma R Bagiire D Mayanja Y Ssali A. Sexual behaviour risk among male regular Partners of Female sex Workers. Adv Glob Heal. (2022) 1:1547913. doi: 10.1525/agh.2022.1547913

[39] Golub SA Fikslin RA Goldberg MH Peña SM Radix A. Predictors of PrEP uptake among patients with equivalent access. AIDS Behav. (2019) 23:1917–24. doi: 10.1007/s10461-018-2376-y, PMID: 30600456 PMC6571035

